# Identification and pathogenicity of *Alternaria* and *Fusarium* species associated with bagged apple black spot disease in Shaanxi, China

**DOI:** 10.3389/fmicb.2024.1457315

**Published:** 2024-09-12

**Authors:** Dandan Ding, Yating Shao, Jirong Zhao, Jinshui Lin, Xiangqian Zhang, Xiukang Wang, Xiangming Xu, Chengnan Xu

**Affiliations:** ^1^Shaanxi Key Laboratory of Research and Utilization of Resource Plants on the Loess Plateau, Yan’an University, Yan’an, China; ^2^Pest and Pathogen Ecology, NIAB East Malling, West Malling, United Kingdom

**Keywords:** apple, black spot disease, *Alternaria* and *Fusarium*, co-infection, pathogenicity

## Abstract

Apple is an economically important fruit crop in northern Shaanxi Province in China. In recent years, a new type of apple black spot disease, “bagged apple black spot disease,” has occurred in the main apple production area of Yan’an City, Shaanxi Province, during the apple ripening season. It seriously affects the appearance quality and commercial value of apples. In this study, 120 isolates recovered from symptomatic apples were identified based on morphological characteristics, pathogenicity, and multilocus sequence analyses of the internal transcribed spacer of ribosomal DNA (rDNA-ITS), translation elongation factor 1-α (*EF1-α*), RNA polymerase II subunit B (*RPB2*), endopolygalacturonase (*endo-PG*), and anonymous region *OPA1-3. Alternaria alternata* was the most abundant species (64%), followed by *Fusarium acuminatum* (36%). Pathogenicity assays were conducted by inoculating them individually and together on detached apples (Venus Golden and Fuji varieties). The results showed that the two fungal species could infect apples individually and together. Co-infection enhanced the disease severity. *F. acuminatum* led to increased severity and speed of disease development compared to *A. alternata*. This is the first report of *Fusarium* and *Alternaria* co-infection causing apple black spot disease worldwide, and the first report of *F. acuminatum* affecting apples. The optimal growth of *A. alternata* occurred at 25–30°C and pH 7; the optimal growth of *F. acuminatum* occurred at 25°C and pH 7. The results of this study can provide a theoretical basis for exploring the occurrence and epidemiology of apple black spot disease and strategies for its control.

## 1 Introduction

Apple is one of the most widely grown and consumed fruits in the world, and China is the world’s largest apple producer, producing about half of the world’s apple supply (Liang et al., 2022). Shaanxi Province is the most important apple-producing area in China, and its cultivation area and yield rank first in the country. Apples are one of the most distinctive and advantageous agricultural products in Shaanxi, and they have strong international competitiveness (Zhou et al., 2021). The Loess Plateau in northern Shaanxi is recognized globally as a high-quality apple-producing area. The apple industry has become one of the fastest growing and most efficient agricultural industries in the region, as well as a major industry that farmers participate in to augment their income (Yan et al., 2019).

Apple bagging is an important technical measure in apple cultivation in China. Bagging fruits can reduce damage caused by diseases and insect pests, reduce pesticide residues on fruits, improve the appearance of fruits, and increase the economic gains. However, with the increased use of fruit bagging, “bagged apple black spot disease,” which causes spots (with various shapes, sizes, and colors) on apple surfaces, collectively referred to as black spot disease involving black necrotic spots (with various shapes, sizes, and colors) on apple surfaces, often occurs during apple harvesting. The disease can cause 5–30% yield losses each year, and up to 80% in severe cases (Li et al., 2013; Wang et al., 2015). The disease strongly affects apple quality and commodity value and has become a major obstacle to the development of China’s apple industry, seriously dampening fruit growers’ enthusiasm for growing apple trees (Hu et al., 2016). The disease is caused by changes in the temperature, humidity, air permeability, and other microecological environmental factors affecting bagged apples (Luan et al., 2019).

Due to the significant differences in recent years in climate conditions in the main apple cultivation areas in China, there are large differences in the pathogen species composition and pathogenicity characteristics of the disease among different areas, as evidenced by pathogen morphology and multi-gene phylogenetic analysis. From 2000 to 2023, a variety of causal fungal species have been reported throughout China. In Gansu Province in 2000, *Cylindrosporium pomi* Brooks and *Phoma pomi* Pass were reported to cause bagged apple black spot disease (based on traditional phytopathogenic fungi morphology plus pathogenicity methods) (Xu and Wei, 2000; Xu et al., 2002). In Shandong Province in 2014, bagged apple black spot disease was divided into four symptom types, which were reported to be caused by *Acremonium sclerotigenum*, *Alternaria tenuissima*, and *Trichothecium roseum* (Wang et al., 2014; Wang et al., 2015). In Guanzhong region of Shaanxi Province in 2016, bagged apple black spot disease was also divided into four symptom types, which were reported to be caused by eight fungal genera, including the taxa *Acremonium* spp., *Alternaria alternata*, and *Trichothecium roseum* (Hou, 2016). In Shaanxi Province in 2019, bagged apple black spot disease were reported to be caused by *Acremonium mali*, *Sarocladium liquanensis*, and *Sarocladium mali* (based on morphology plus phylogenetic analysis involving ITS, ACT, LSU and BT2) (Hou et al., 2019). In Shaanxi Province in 2018, *Alternaria malicola* sp. nov. was discovered as a new species affecting apple fruits (Dang et al., 2018). In Hebei Province in 2021, bagged apple black spot disease occurred widely and there were four symptom types. They were reported to be caused by five fungal genera, with *Trichothecium roseum* being dominant, followed by *Acremonium sclerotigenum*, and *Alternaria tenuissima* (based on morphology plus sequence analysis involving large subunit (*LSU*) and *OPA10-2*) (Meng et al., 2021). In conclusion, the pathogens of bagged apple black spot disease differ greatly by region in China.

From 2020 to 2023, the incidence of bagged apple black spot disease was >75% in some orchards in Baota District and Yichuan County of Yan’an City, northern Shaanxi. *Alternaria* spp. and *Fusarium* spp. were the putative causal agents. Due to the dramatic differences in climate conditions across the mountainous fruit cultivation areas of the Loess Plateau in northern Shaanxi, the symptoms and pathogen composition of the disease are very complex, and clarifying the dominant pathogens is key to effective scientific control of the disease. Therefore, it was necessary to identify and analyze the pathogens causing bagged apple black spot disease in Baota District and Yichuan County of Yan’an City, northern Shaanxi. The objectives of this study were (1) to investigate the incidence of bagged apple black spot disease in Baota District and Yichuan County of Yan’an City, northern Shaanxi, and to identify the pathogenic species by morphological and multi-gene sequence analysis; (2) to clarify the pathogenicity of the pathogen on the main cultivated apple varieties; and (3) to clarify the effects of temperature and pH on the mycelial growth of the main pathogens.

## 2 Materials and methods

### 2.1 Sample collection and fungal isolation

Bagged apples with black spot disease were collected from three orchards in (1) Baota District and (2) Yichuan County of Yan’an City, Shaanxi Province, during apple harvesting from September to October 2023. The main varieties planted in the orchards were Fuji and Venus Gold. Apples fruits with typical symptoms of bagged apple black spot disease were collected with plastic bags (labeled with the place, time, and number of samples), and brought back to the laboratory.

The apples were disinfected with 75% alcohol for 2 min, rinsed twice with sterile water, and naturally air-dried. They were then cut with a sterile blade to obtain infected apple tissue samples (0.5 × 0.5 × 0.5 cm) from the margins of black spot lesions, which were transferred to potato dextrose agar (PDA) plates supplemented in advance with 50 μg/mL streptomycin sulfate. The cultures were incubated for 3–5 days in an incubator at 25°C in the dark. Hyphal tips from the developing fresh mycelium were transferred to fresh PDA plates for purification. Based on the morphological characteristics of the colonies and conidia, the isolates were divided into two groups: *Alternaria* and *Fusarium*. All putative *Alternaria* and *Fusarium* isolates were subcultured on PDA plates for single spore purification. The representative isolates were transferred to PDA plates, and putative *Fusarium* isolates were also transferred to carnation leaf agar (CLA) medium plates. The colonies were cultured in a constant-temperature incubator at 25°C for 5–20 days. A total of 120 isolates were obtained and stored at 4°C. The isolates were identified to species level based on the morphology and molecular phylogenetic analysis.

### 2.2 Morphological characterization

The colony diameters were assessed daily, and the mean daily colony growth rate (mm/day) was calculated. The morphological characteristics of the colonies and conidia were observed and photographed under a microscope daily. Fifty conidia were randomly selected to measure their length and width. The isolates were identified based on the morphological characteristics of colonies and conidia in previous studies (Leslie and Summerell, 2006; Simmons, 2007).

### 2.3 DNA extraction, PCR amplification, and phylogenetic analysis

Representative four *Alternaria* isolates and seven *Fusarium* isolates in this study were inoculated on PDA medium and cultured at 26°C in the dark for 7 days. A sample of the mycelium (50 mg) of each isolate was collected from the surface of the PDA medium and then transferred to a 1.5-ml centrifuge tube. The genomic DNA of the mycelium was then extracted using the cetyltrimethylammonium bromide (CTAB) method (Zhou et al., 2011), dissolved in ddH_2_O, and stored at −20°C. Internal transcribed spacer of ribosomal DNA (rDNA-ITS), translation elongation factor 1-α (*EF1-α*), endopolygalacturonase (*endo-PG*), anonymous region *OPA1-3*, and RNA polymerase II subunit B (*RPB2*) gene fragments were PCR amplified and sequenced using the following primers (Table 1) (Shanghai Bioengineering Co., Ltd.): ITS1/ITS4, EF1-728F/EF1-986R, PG3/PG2b, OPA1-3 L/OPA1-3R, and RPB2-5F2/FRPB2-7cR. The 25 μL PCR system contained 12.5 μL 2× Taq enzyme, 1.5 μL DNA, 0.5 μL upstream primer, 0.5 μL downstream primer, and 10 μL ddH_2_O. The amplified PCR products were assessed by 1% agarose gel electrophoresis and sent to Sangon Biotech (Shanghai, China) for sequencing. Our ITS, *EF1-α*, *RPB2*, *endo-PG*, and *OPA1-3* sequences were compared to related sequences in the NCBI database by BLAST, and our sequences were submitted to GenBank (accession numbers are listed in Table 2).

**Table 1:** Summary of published universal primers used for *Fusarium* and *Alternaria* species determination associated with bagged apple black spot in Shaanxi, China.

Gene/locus	Primer name	Primer sequences (5′-3′)*	PCR amplication on procedures	References
ITS	ITS1	TCCGTAGGTGAACCTGCGG	94°C 5 min; 30 cycles of 94°C 1 min, 55°C 30 s, 72°C 1 min; 72°C 10 min	Woudenberg et al. (2014)
	ITS4	TCCTCCGCTTATTGATATGC		
TEF1-α	EF1-728F	CATCGAGAAGTTCGAGAAGG	95°C 3 min; 35 cycles of 94°C 30 s, 55°C 30 s, 72°C 1 min; 72°C 10 min	Carbone and Kohn (1999)
	EF1-986R	TACTTGAAGGAACCCTTACC		
endoPG	PG3	TACCATGGTTCTTTCCGA	94°C 5 min; 35 cycles of 94°C 45 s, 56°C 45 s, 72°C 45 s; 72°C 7 min	Andrew et al. (2009)
	PG2b	GAGAATTCRCARTCRTCYTGRTT		
OPA1-3	OPA1-3 L	CAGGCCCTTCCAATCCAT	94°C 5 min; 35 cycles of 94°C 45 s, 58°C 45 s, 72°C 45 s; 72°C 7 min	Peever et al. (2004)
	OPA1-3R	AGGCCCTTCAAGCTCTCTTC		
RPB2	RPB2-5F2	GAYGAYMGWGATCAYTTYGG	94°C 5 min; 5 cycles of 94°C 45 s, 60°C 45 s, 72°C 2 min; 5 cycles of 94°C 45 s, 58°C 45 s, 72°C 2 min; 30 cycles of 94°C 45 s, 54°C 45 s, 72°C 2 min; 72°C 7 min	Woudenberg et al. (2014)
	fRPB2-7cR	CCCATRGCTTGYTTRCCCAT		

*Y = T or C; M = A or C; W = A or T; R = A or G.

**Table 2:** *Alternaria* and *Fusarium* isolates obtained in this study and downloaded from GenBank with accession numbers used for phylogenetic analyses.^a^

			GenBank accession No.				
Isolate	Species	origin	ITS	EF1-α	RPB2	endoPG	OPA1-3
**WHWNSHJ4**	***F. acuminatum***	**Baota**	**PP336551**	**PP351904**	**–**	**–**	**–**
**WHWNSHJ5**	***F. acuminatum***	**Baota**	**PP336552**	**PP351905**	**PP351911**	**–**	**–**
**WHWNSHJ1**	***F. acuminatum***	**Baota**	**PP336553**	**PP351906**	**PP351912**	**–**	**–**
**YICASTK7**	***F. acuminatum***	**Yichuan**	**PP336554**	**PP351907**	**PP351913**	**–**	**–**
**YICASTK4**	***F. acuminatum***	**Yichuan**	**PP336555**	**PP351908**	**–**	**–**	**–**
**YICASTK3**	***F. acuminatum***	**Yichuan**	**PP336556**	**PP351909**	**–**	**–**	**–**
**YICASTK12**	***F. acuminatum***	**Yichuan**	**PP336557**	**PP351910**	**PP351914**	**–**	**–**
NL19-077002^*^	*F. acuminatum*	Netherlands	MZ890557	MZ921910	MZ921779	**–**	**–**
F201136	*F. acuminatum*	China	KM527098	KM527106	KM520372	**–**	**–**
CYF017	*F. proliferatum*	China	MG384385	MG674276	MK027419	**–**	**–**
CYF035	*F. proliferatum*	China	MG384386	MG674279	MK027420	**–**	**–**
CBS 101427^*^	*F. solani*	USA	EU329691	DQ246834	**–**	**–**	**–**
NRRL 29132^*^	*F. solani*	Germany	DQ094388	DQ246915	**–**	**–**	**–**
P55HS	*F. tricinctum*	Poland	**–**	MZ078998	MZ078959	**–**	**–**
CBS 119173^*^	*F. graminearum*	Poland	**–**	KT855178	KT855204	**–**	**–**
NRRL 20697^*^	*F. equiseti*	USA	GQ505683	GQ505594	JX171595	**–**	**–**
CBS 632.76^*^	*F. lunatum*	Germany	EU926224	EU926291	–	**–**	**–**
**SWHBS4-2**	***A. alternata***	**Baota**	**PP346356**	**PP351930**	**PP351921**	**PP351915**	**PP376078**
**YICWNSHJ9**	***A. alternata***	**Yichuan**	**PP346360**	**PP351933**	**PP351925**	**PP351917**	**PP376082**
**YICWNSHJ7**	***A. alternata***	**Yichuan**	**PP346361**	**PP351934**	**PP351926**	**PP351918**	**PP376083**
**YICASTK5**	***A. alternata***	**Yichuan**	**PP346363**	**PP351936**	**PP351922**	**PP351920**	**PP376085**
CBS 106.24^*^	*A. alternata*	USA	**–**	KP125073	KP124766	AY295020	**–**
CBS 104.26	*A. alternata*	Unknown	**–**	KP125074	KP124767	KP123995	**–**
CH-37	*A. alternata*	Italy	**–**	**–**	OP899773	OP899733	**–**
9-1	*A. alternata*	China	**–**	**–**	**–**	OK428544	**–**
A10	*A. alternata*	China	**–**	**–**	**–**	MN894680	
CBS 118486^*^	*A. iridiaustralis*	Australia	–	KP125214	KP124905	KP124140	**–**
CBS 878.95	*A. jacinthicola*	Mauritius	–	KP125216	KP124907	KP124142	**–**
CBS 133751^*^	*A. jacinthicola*	Mali	**–**	KP125217	KP124908	KP124143	**–**
CBS 113.35	*A. longipes*	Unknown	**–**	KP125219	KP124910	KP124145	**–**
CBS 539.94	*A. longipes*	USA	**–**	KP125220	KP124911	KP124146	**–**
CBS 118809^*^	*A. alstroemeriae*	Australia	**–**	KP125072	KP124765	KP123994	**–**
CBS 107.38^*^	*A. burnsii*	India	**–**	KP125198	KP124889	KP124124	**–**
CBS 489.92^*^	*A. eichhorniae*	India	**–**	KP125204	KP124895	KP124130	**–**
EGS 90–0512	*A. gaisen*	Japan	**–**	KC584658	KC584399	AY295033	**–**
CBS 102605^*^	*A.arborescens*	USA	–	KC584636	KC584377	AY295028	**–**
CBS 109730	*A.arborescens*	USA	–	KP125177	KP124869	KP124103	**–**

a All the ex-type isolates used in this study are marked by an asterisk (*). The isolates in this study are indicated in bold font.

Based on a report by Woudenberg et al., the *EF1-α*, *RPB2*, and *endo-PG* sequences of 6 standard *Alternaria* isolates were downloaded from the NCBI database (Woudenberg et al., 2015). Based on reports by Crous et al. and Zhou et al., rDNA-ITS, *EF1-α*, and *RPB2* sequences of 2 standard *Fusarium* isolates were downloaded from the NCBI database (Zhou et al., 2016; Crous et al., 2021a). The obtained sequences were edited using Bio-Edit software, and multiple sequence alignment was performed using Clustal X 1.83. The sequences of our four representative *Alternaria* isolates and seven representative *Fusarium* isolates were compared to those of the standard isolates in MEGA 7.0. Maximum-likelihood phylogenetic trees were constructed, and confidence was evaluated using the bootstrap method with 1,000 replications. According to the results of phylogenetic tree analysis and sequence alignment, the species of the representative isolates obtained in this study were determined.

### 2.4 Temperature and pH experiments

As all the *Alternaria* and *Fusarium* isolates in the two respective groups exhibited similar growth characteristics, representative isolates *A. alternata* YICASTK5 and *F. acuminatum* WHWNSHJ1 were selected to evaluate the effects of temperature and pH on colony growth on PDA medium. A 5-mm-diameter mycelial plug of each isolate from the edge of a 7-day-old colony was placed in the center of a 90-mm-diameter PDA plate and incubated in the dark. Temperatures ranged from 5°C to 40°C at 5°C intervals (5°C, 10°C, 15°C, 20°C, 25°C, 30°C, 35°C, and 40°C). PDA medium was adjusted to 3.0, 4.0, 5.0, 6.0, 7.0, 8.0, 9.0, 10.0, and 11.0 using 0.1 mol/L sodium hydroxide (0.1 M NaOH) and 0.1 mol/L hydrochloric acid (0.1 M HCl). The colony diameters were measured daily. The experiments involved three replicates and were repeated twice. GraphPad Prism 9.0 was used for data analysis and visualization.

### 2.5 Pathogenicity assays

The representative isolates *A. alternata* YICASTK5 and *F. acuminatum* WHWNSHJ1 obtained in this study underwent pathogenicity assays. There were three treatments: (1) *Alternaria* inoculation; (2) *Fusarium* inoculation; and (3) co-inoculation with both fungi. Healthy, mature, detached Fuji and Venus Golden apples of the same size were surface sterilized with 75% alcohol for 2 min and then washed with sterile water twice. A 5-mm-diameter PDA plug containing mycelia was taken from a 5-day-old colony and used to inoculate an apple that had been punctured at the inoculation site with a sterile syringe needle to a depth of 2 mm. The co-inoculation method comprises the following steps: representative isolates *A. alternata* YICASTK5 and *F. acuminatum* WHWNSHJ1 were cultured in the same PDA petri dish at 25°C. When the mycelium of two strains grow together in the center of PDA petri dish, 5-mm-diameter PDA plug containing mycelia for both species were taken from the junction of mycelium for inoculation. The inoculated apples were placed in a plastic box (25 × 15 × 10 cm) and left for 10 days at 25°C and 100% relative humidity. There were three inoculation sites per apple and three apples per treatment, and the experiment was repeated three times per treatment. Pathogenicity was determined by the necrotic lesion diameter at 3 days after inoculation. To fulfill Koch’s postulates, small pieces of infected apple tissues were plated on PDA medium to reisolate the fungal isolates, which were reidentified based on morphological characterization and DNA sequences.

### 2.6 Statistical analyses

The statistical analysis was performed using GraphPad Prism 9.0 software. Lesion diameters measured in the pathogenicity test were subjected to the analysis of variance (ANOVA) and the mean values were separated by Tukey test (*p* = 0.05).

## 3 Results

### 3.1 Field disease symptoms and pathogen isolation

From the end of September to the beginning of October 2023, bagged apple black spot disease was investigated in three commercial apple orchards in (1) Baota District and (2) Yichuan County of Yan’an City, Shaanxi Province. The management level is medium and both Fuji and Venus Gold varieties are grown there. Black spots on the bagged apples in the three orchards occurred throughout the study period. The incidence was 30 and 75% for Fuji and Venus Golden apples, respectively.

**Figure 1:**
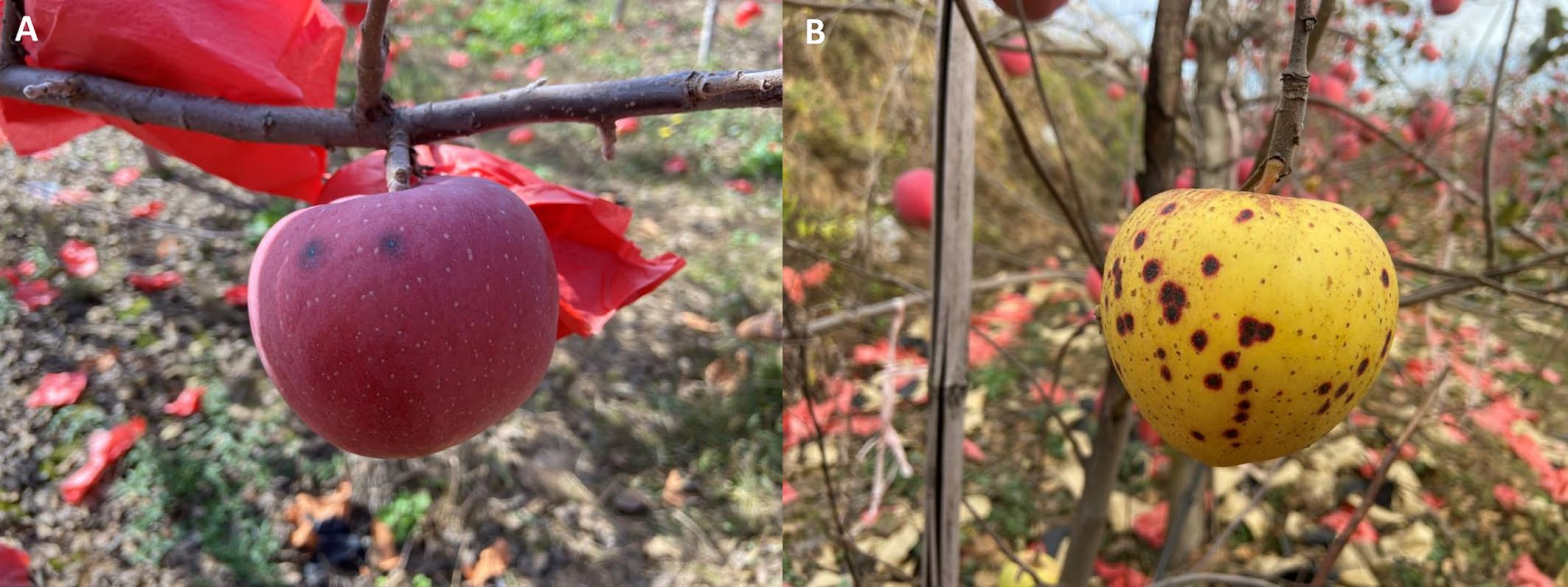
Field symptoms of bagged apple black spot disease caused by *Alternaria alternata* and *Fusarium acuminatum* in Shaanxi Province, China. Typical symptoms on **(A)** Fuji and (**B)** Venus Golden apples.

The main symptoms of apple black spots are as follows: small black spots (approximately round, with a diameter of about 2–5 mm and a lenticel at the center) on the surface of the apple are formed as it reaches maturity. The pericarp around each spot is necrotic, forming a red or black halo (Figures 1A,B). The spots are unevenly distributed on the surface and the lesions do not expand, but the apples completely lose their commercial value.

A total of 51 diseased apples (25 Fuji and 26 Venus Gold apples) were collected from the three orchards, with varying disease severity. A total of 120 suspected pathogenic isolates were obtained, involving 77 (64%) *Alternaria* isolates and 43 (36%) *Fusarium* isolates (*Alternaria* was the dominant genus). For the Fuji apples, 26 *Alternaria* isolates and 23 *Fusarium* isolates were isolated (*Alternaria* was the dominant genus). For the Venus Golden apples, 51 *Alternaria* isolates and 20 *Fusarium* isolates were isolated (*Alternaria* was the dominant genus). These isolates were stored at the Phytopathology Research Laboratory of Yan’an University.

### 3.2 Morphological characterization

Based on morphological characteristics (colony morphology and conidial characteristics) and phylogenetic analysis, the 120 isolates were divided into two groups: 77 isolates in group A (identified as *Alternaria* spp.) and 43 isolates in group B (identified as *Fusarium* spp.). The morphological characteristics of isolates in each group were consistent, so *Alternaria* isolate YICASTK5 and *F. acuminatum* WHWNSHJ1 were selected as the main representative isolate, inoculated on PDA medium, and cultured in the dark in a constant-temperature incubator at 25°C. The growth status of the isolates was observed and recorded daily.

**Figure 2:**
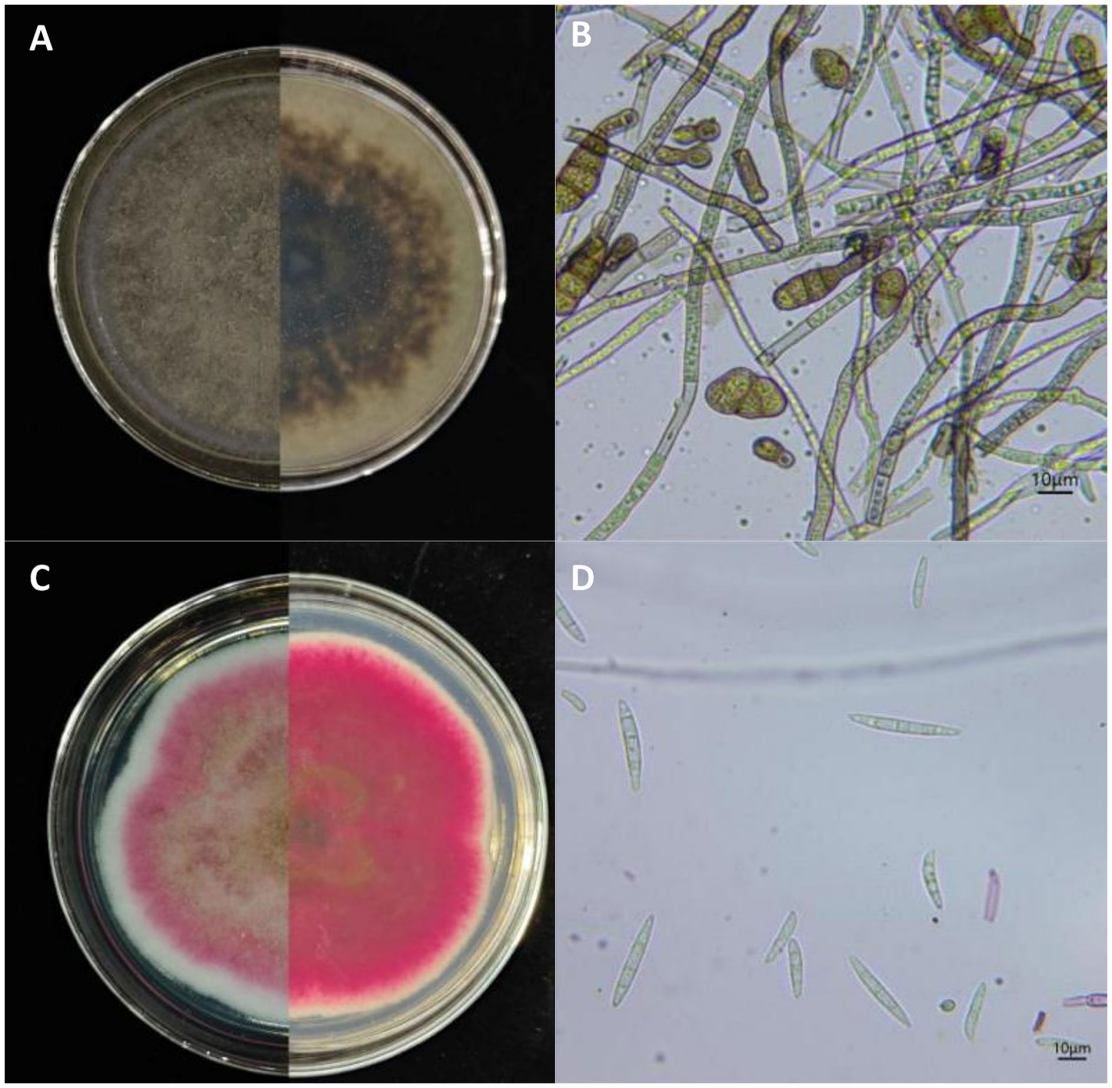
Morphological characteristics of *Alternaria alternata* and *Fusarium acuminatum* isolated from apples with bagged apple black spot disease in Shaanxi Province, China. **(A)** Front and reverse of colonies cultured on PDA medium for 7 days and **(B)** conidia of *A. alternata* YICASTK5 (representative isolate). Scale bar: D = 10 μm. **(C)** Front and reverse of colonies cultured on PDA for 15 days and **(D)** conidia of *F. acuminatum* WHWNSHJ1 (representative isolate). Scale bar: D = 10 μm.

Group A: On PDA medium, the colony is olive green to grayish brown with densely packed hyphae on the front side and a dark gray reverse side (Figure 2A), with a growth rate of 8.50 mm/day on PDA medium. The conidiophores are brown, either single or branched, and produce numerous short conidial chains. The conidia are ovoid or ellipsoid, with 1 to 5 transverse septa and 0 to 2 oblique septa, and a size of (6.58 to 27.73) μm × (5.13 to 16.18) μm (*n* = 50) (Figure 2B). Morphological characteristics were consistent with description of *A. alternata* (Simmons, 2007).

Group B: On PDA medium, colony grew slowly, with a growth rate of 4.30 mm/day on PDA medium. Isolate formed abundant white aerial mycelium, then became floccose with rose pigmentation, and developed a brownish tinge in the center and grayish rose at the periphery on PDA after 15 days’ inoculation. The reverse side of the colony was red to burgundy (Figure 2C). On carnation leaf agar, macroconidia with 3 to 5 septa were abundant, relatively slender, curved to lunate, and measured (24.65 to 55.40) μm × (3.40 to 5.95) μm (*n* = 50) (Figure 2D). Microconidia were oval, with 0 to 1 septa. Chlamydospores were globose with a smooth outer wall in chains or single. Morphological characteristics were consistent with description of *F. acuminatum* (Leslie and Summerell, 2006).

### 3.3 Molecular characterization

Five gene fragments (ITS, *EF1-α*, *RPB2*, *endo-PG*, and *OPA1-3*) commonly used for *Alternaria* spp. and *Fusarium* spp. identification were selected for PCR and sequencing to identify the four representative *Alternaria* isolates and seven representative *Fusarium* isolates. The sequences were submitted to GenBank (accession numbers are listed in Table 2). Based on ITS, *EF1-α*, *RPB2*, *endo-PG*, and *OPA1-3*, our four representative *Alternaria* isolates exhibited 99–100% similarity to the nucleotide sequences of representative *Alternaria alternata* isolates from GenBank (using BLAST searches). Based on ITS, *EF1-α*, and *RPB2*, our seven representative *Fusarium* isolates exhibited 99–100% similarity to the nucleotide sequences of representative published *Fusarium acuminatum* isolates from GenBank (using BLAST searches).

**Figure 3:**
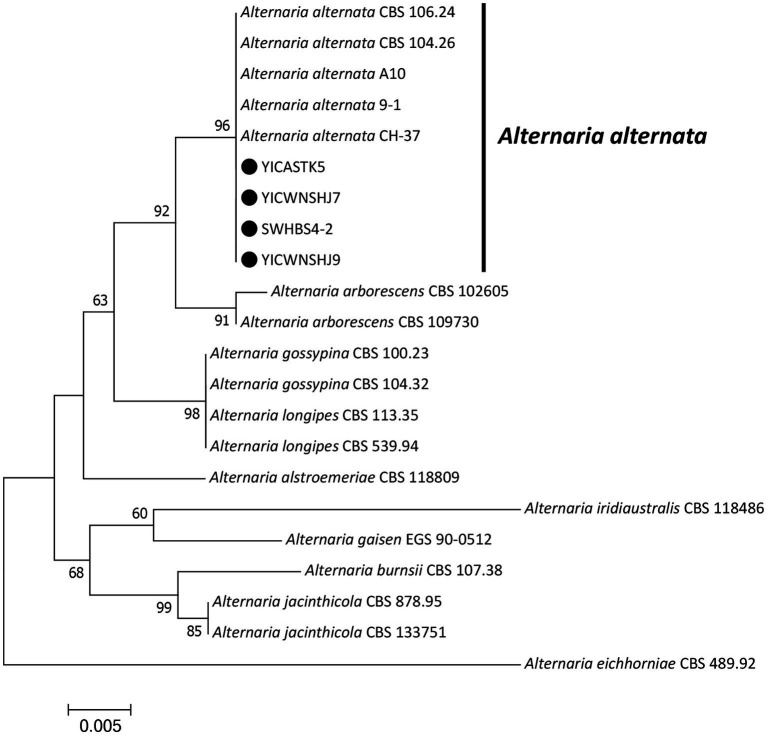
Maximum-likelihood phylogenetic tree based on translation elongation factor 1-α (*EF1-α*), RNA polymerase II subunit B (*RPB2*), and endopolygalacturonase (*endo-PG*) gene sequences of our four representative *Alternaria alternata* isolates associated with bagged apple black spot disease in China (indicated by ●). Related *Alternaria* species were downloaded from GenBank. Support values at nodes represent RAxML bootstrap percentages ≥60%.

**Figure 4:**
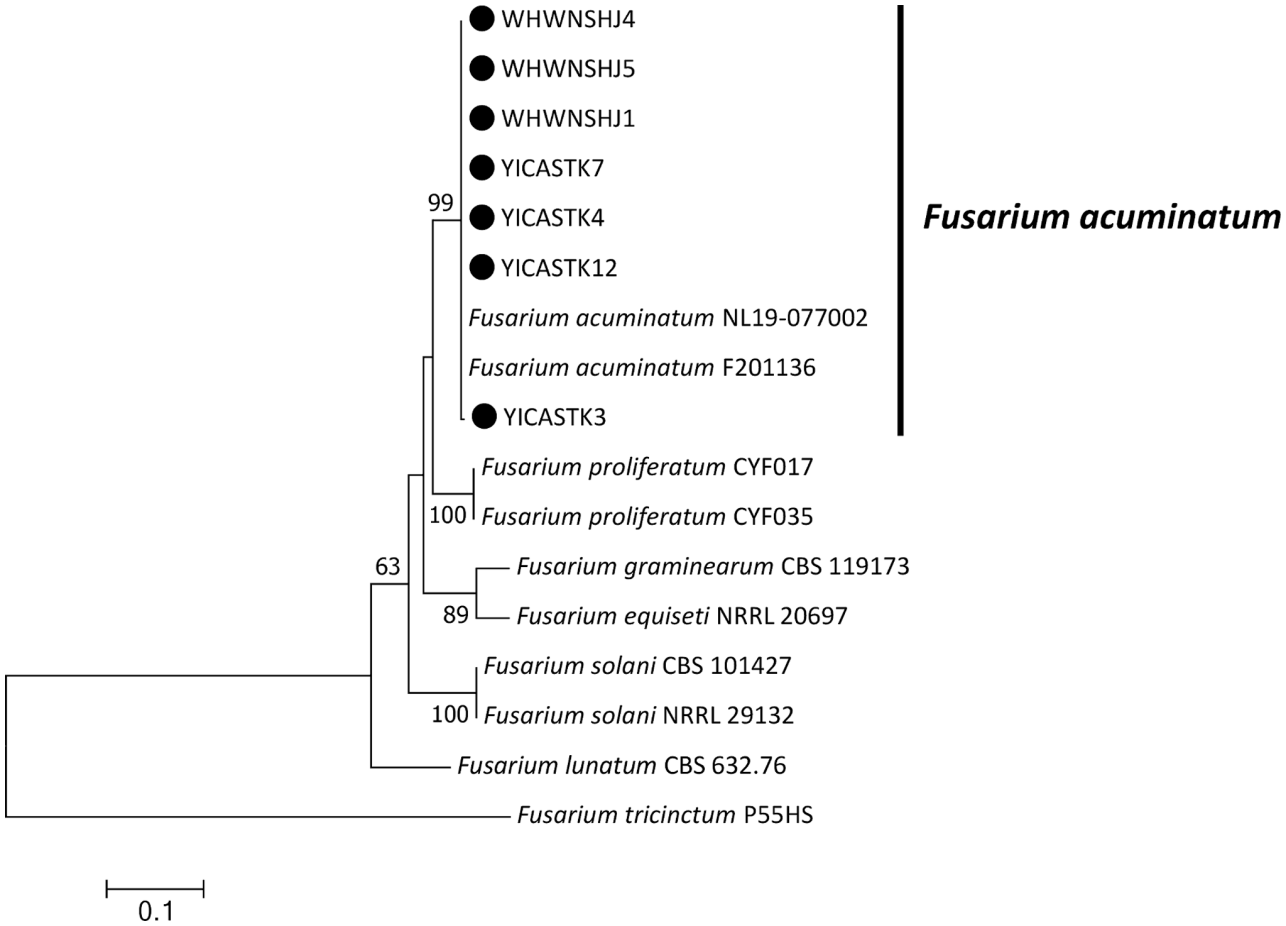
Maximum-likelihood phylogenetic tree based on internal transcribed spacer of ribosomal DNA (rDNA-ITS), translation elongation factor 1-α (*EF1-α*), and RNA polymerase II subunit B (*RPB2*) gene sequences of our seven representative *Fusarium acuminatum* isolates associated with bagged apple blackspot disease in China (indicated by ●). Related *Fusarium* species were downloaded from GenBank. Support values at nodes represent RAxML bootstrap percentages ≥60%.

Maximum-likelihood phylogenetic trees were constructed in MEGA 7.0 for our four *Alternaria* isolates (based on *EF1-α*, *RPB2*, and *endo-PG*) (Figure 3) and our seven *Fusarium* isolates (based on ITS, *EF1-α*, and *RPB2*) (Figure 4) plus standard *Alternaria* and *Fusarium* isolates sequences, respectively, from GenBank. Our *Alternaria* isolates clustered in the same branch as *A. alternata* CBS104.26, CBS106.24, CH-37, A10, and 9–1 (their phylogenetic relationships were the closest), and the branch support rate was 96%. Our *Fusarium* isolates clustered in the same branch as *F. acuminatum* NL19-077002 and F201136 (their phylogenetic relationships were the closest), and the branch support rate was 99%.

According to the morphological and molecular characterization, the main associated isolates of bagged apple black spot disease in Baota District and Yichuan County of Yan’an City, Shaanxi Province, were *Alternaria alternata* and *Fusarium acuminatum*.

### 3.4 Effects of temperature and pH on mycelial growth

#### 3.4.1 Effects of temperature

**Figure 5:**
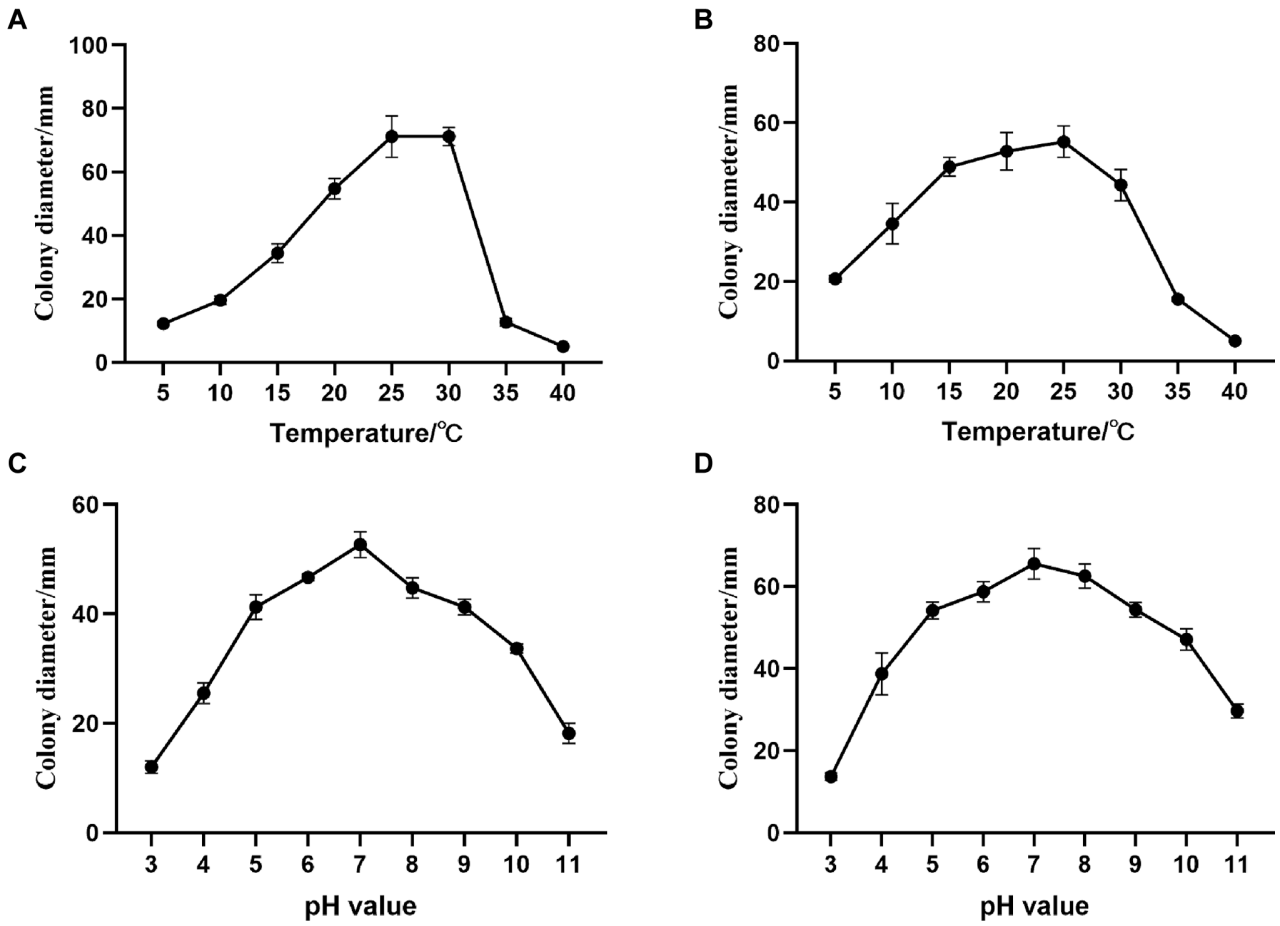
Effects of temperature and pH on *Alternaria alternata* and *Fusarium acuminatum* mycelial growth on PDA medium. Changes in mycelial growth of **(A)** *A. alternata* YICASTK5 with temperature, **(B)** *F. acuminatum* WHWNSHJ1 with temperature, **(C)** *A. alternata* YICASTK5 with pH, and **(D)** *F. acuminatum* WHWNSHJ1 with pH.

*A. alternata* YICASTK5 mycelia grew at 5–35°C but was strongly influenced by temperature (Figure 5A). It grew fastest at 25°C and 30°C, did not grow at 40°C, and grew optimally at 25–30°C.

*F. acuminatum* WHWNSHJ1 mycelia grew at 5–35°C (Figure 5B). The mycelial growth rate gradually increased from 5°C to 25°C and rapidly decreased from 25°C to 35°C. It did not grow at 40°C and grew optimally at 25°C.

#### 3.4.2 Effects of pH

*A. alternata* YICASTK5 mycelia grew at pH 3.0–11.0 (Figure 5C), but it varied significantly, with dense, vigorous. The mycelia grew fastest at pH 7 and slower at pH 3 and 11. Thus, it grew best in a neutral environment.

*F. acuminatum* WHWNSHJ1 mycelia grew at pH 3.0–11.0 (Figure 5D), but it varied significantly, with dense, vigorous, and fastest growth at pH 7, and slower growth at pH 3 and 11. Thus, it grew best in a neutral environment.

### 3.5 Pathogenicity assays

The representative isolates *A. alternata* YICASTK5 and *F. acuminatum* WHWNSHJ1 were inoculated into Fuji and Venus Golden apples. After 3 days, black spots appeared on the apple surfaces, which was basically consistent with the field disease symptoms. The incidence rate was 100%.

**Figure 6:**
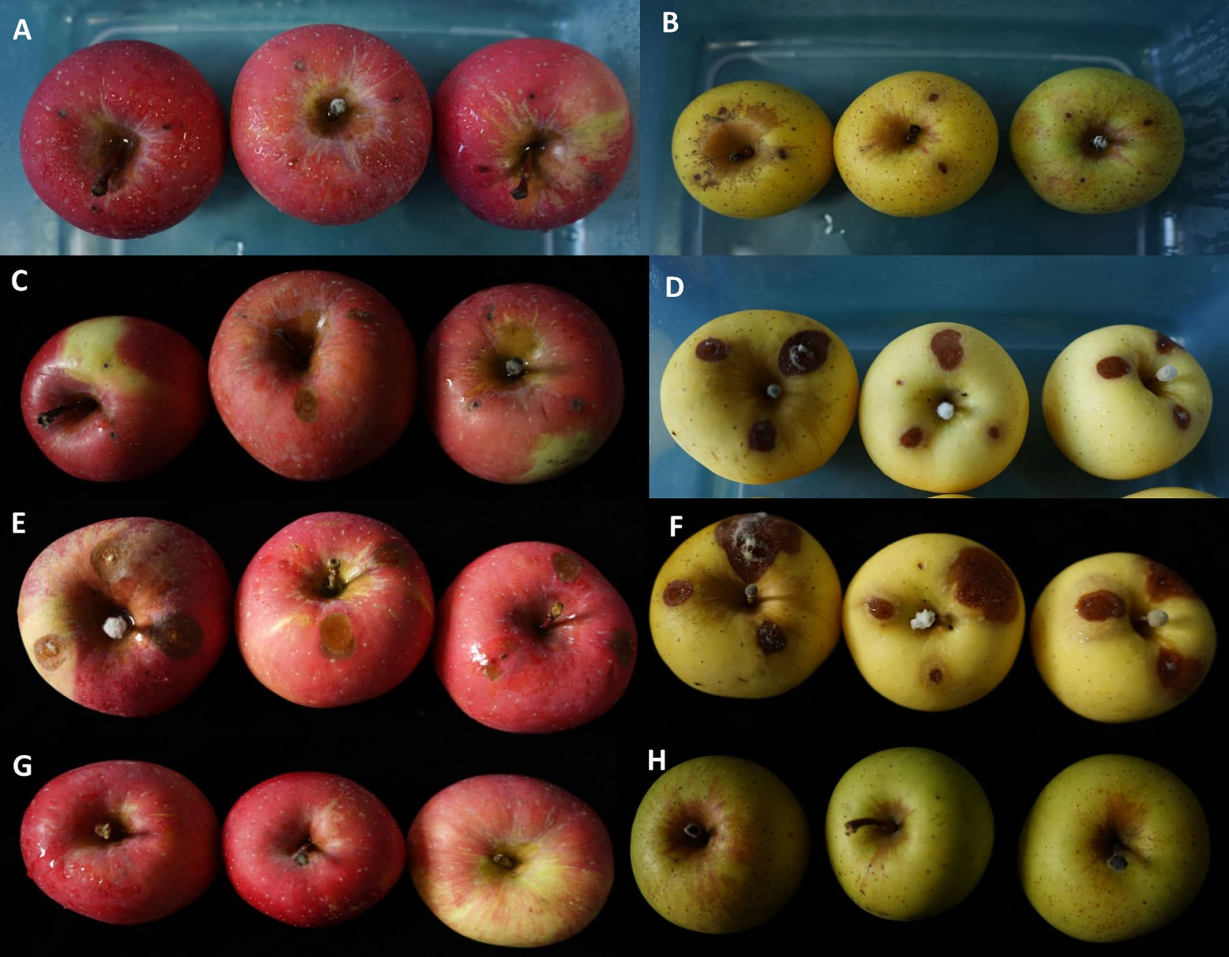
Symptoms on Fuji and Venus Golden apples inoculated with *Alternaria alternata* and *Fusarium acuminatum* at 7 dpi. *A. alternata* YICASTK5 on **(A)** Fuji and **(B)** Venus Golden apples. *F. acuminatum* WHWNSHJ1 on **(C)** Fuji and **(D)** Venus Golden apples. *A. alternata* and *F. acuminatum* co-inoculation on **(E)** Fuji and **(F)** Venus Golden apples. **(G)** Control Fuji apples. **(H)** Control Venus Golden apples.

**Figure 7:**
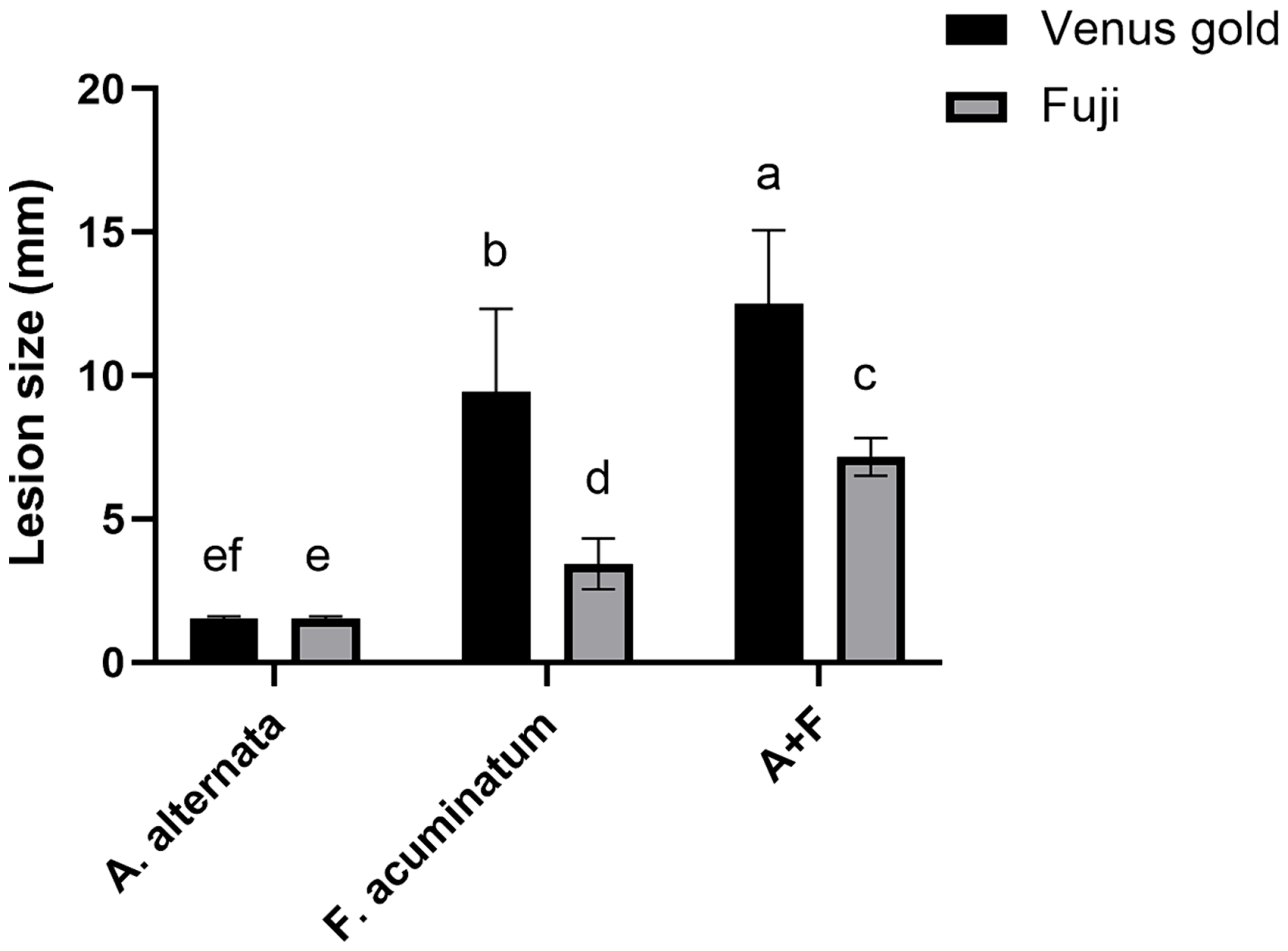
*Alternaria alternata* YICASTK5 and *Fusarium acuminatum* WHWNSHJ1 lesion sizes on Fuji and Venus Golden apples at 7 dpi. Different letters above error bars indicate a significant difference (*p* < 0.05).

At 7 days after *A. alternata* inoculation, the mean lesion diameter was 1.5 mm in both apple varieties (it did not expand further after 7 days) (Figures 6A,B). At 7 days after *F. acuminatum* inoculation, the mean lesion diameter was 3.44 mm in Fuji apples and 9.44 mm in Venus Golden apples (Figures 6C,D). At 7 days after co-inoculation of *A. alternata* and *F. acuminatum*, the mean lesion diameter was 7 mm in Fuji apples and 13.5 mm in Venus Golden apples (Figures 6E,F). Co-inoculation had the strongest impact on both apple varieties, with the lesions expanding the fastest (Figures 6E,F). The control-inoculated apples were not diseased (Figures 6G,H). There were significant differences in lesion size among the three inoculation treatments, with co-inoculation having the strongest effect, *F. acuminatum* inoculation having a moderate effect, and *A. alternata* inoculation having the weakest effect (Figure 7).

Each pathogen was reisolated from apples inoculated with each pathogen by conventional tissue isolation methods. Based on morphological identification, the reisolated pathogens were the same species as the inoculated species. Thus, it was verified that *A. alternata* and *F. acuminatum* were the main pathogens causing bagged apple black spot disease.

## 4 Discussion

Bagged apple black spot disease is becoming an important disease in the Chinese apple industry. In particular, it is a serious problem that needs to be solved for apple growers in the Loess Plateau of northern Shaanxi Province. It has a large impact on the appearance of apples, seriously affecting the percentage of quality apples and commodity value. In this study, *A. alternata* and *F. acuminatum* were identified as causal agents of this disease in the northern areas of Shaanxi Province: (1) Baota District and (2) Yichuan County of Yan’an City. *A. alternata* was dominant, which may be related to the introduction and cultivation of Venus Golden apples (for the Venus Golden apples, 20 *Fusarium* isolates vs. 51 *Alternaria* isolates were isolated) and the arid climate in the Loess Plateau of northern Shaanxi. To our knowledge, this is the first report in the world on *Alternaria* and *Fusarium* co-infection of apples, and this is also the first report on *F. acuminatum* causing apple disease in the world.

Apple is an economically important fruit crop that is widely cultivated in China. Due to the differential effects of climate change in different regions, bagged apple black spot disease can be caused by a variety of pathogens. *Acremonium sclerotigenum*, *Alternaria tenuissima*, and *Trichothecium roseum* have been widely reported in Hebei provinces (Meng et al., 2021). *A. tenuissima* and *T. roseum* are the dominant pathogens causing bagged apple black spot disease in Shandong, Shaanxi, and western Liaoning (Hao et al., 2004; Zhou et al., 2012; Wang et al., 2014). The genus of *Acremonium*, *Alternaria*, *Sarocladium*, and *Trichothecium* exhibited weak pathogenicity after inoculation of punctured apples (Wang et al., 2014; Hou, 2016; Meng et al., 2021). Our results on *A. alternata* inoculation of punctured Fuji and Venus Golden apples were similar. Thus, these pathogens could be considered as opportunistic, weak pathogens, which can only infect the surface of apples under suitable conditions. However, most of the previous studies did not carry out inoculation tests on Venus Golden apples, while we studied both Fuji and Venus Golden apples. Our inoculation tests after puncturing the apples showed that both *A. alternata* and *F. acuminatum* are pathogenic to both Fuji and Venus Golden apples, though *A. alternata* was only weakly pathogenic to both varieties.

Pathogencity tests showed that *A. alternata* and *F. acuminatum* have variations in the disease progression on detached apple fruits. *F. acuminatum* infects fruits and develops symptoms more quickly than *A. alternate.* Additionally, compared to single infection of these two species, *Alternaria* and *Fusarium* co-inoculation significantly increased the pathogenicity. Based on the results above, it is evident that apple black spot disease is a complex disease that might be caused by different fungal species. These different fungal taxa may have varying impacts on apple fruits. Our findings suggest that multiple fungi causing a primary infection in an apple orchard during apple ripening can increase the disease complexity. Researchers in Australia and Israel have found that many *Alternaria* isolates can concurrently cause diseases on apple leaves and fruits, and cross-pathogenicity assays have shown that pathogenic *Alternaria* isolates do not exhibit specificity regarding apple leaves or fruits (Harteveld et al., 2014; Gur et al., 2017). Apple orchards are complex ecosystems and identifying the primary infection source of apple diseases is very important for effective scientific disease control. We speculate that the pathogens isolated from apple fruits surfaces in this study may originate from apple leaf fungal infections.

The genus *Alternaria* was established by Nees von Esenbeck in 1816. It contains many endophytic, saprophytic, and pathogenic species, and *A. tenuis* is the representative species. Traditional *Alternaria* taxonomic identification is mostly based on morphological characteristics (Simmons, 1967), but this is often affected by conditions such as medium composition, culture environment, and culture duration (Thomma, 2003). Therefore, molecular techniques have been widely used to aid morphological identification of *Alternaria* (Kusaba and Tsuge, 1995; Peever et al., 2004). Gene segments such as *EF1-α*, *RPB2*, *endo-PG*, *OPA1-3*, *CAL*, and *TUB* have been widely used in molecular phylogenetic analyses of *Alternaria* species (Andrew et al., 2009; Lawrence et al., 2013; Woudenberg et al., 2014; Woudenberg et al., 2015; Zheng et al., 2015). Recent studies have shown that multiple *Alternaria* microspore species can co-infect a variety of fruit trees, including apple, blueberry, and pomegranate (Gao et al., 2013; Ntasiou et al., 2015; Zhu and Xiao, 2015; Luo et al., 2017). In this study, three gene fragments combined phylogenetic analyses (*EF1-α*, *RPB2* and *endo-PG*) accurately distinguished the *Alternaria* isolates.

The genus *Fusarium* Link is widely distributed in nature, and it is one of the most important phytopathogenic fungi. Symptoms (including rhizome rot, fruit rot, wilting, and leaf spotting) caused by *Fusarium* species on various plants can lead to severe economic losses (Leslie and Summerell, 2006; Wang et al., 2022). In recent years, disease caused by *Fusarium* has gradually become a severe problem in global apple cultivation. *Fusarium proliferatum* was confirmed as the dominant pathogen of apple replant disease in China (based on phylogenetic analysis involving six gene loci) (Duan et al., 2022). *Fusarium tricinctum* was first reported as a new pathogen causing apple dieback in Gansu, China (Zhang et al., 2022). *Fusarium fujikuroi* was first identified as the pathogen of apple heart rot in India (based on morphology, molecular analysis, and pathogenicity) (Watpade et al., 2024). *Fusarium decemcellulare* was first reported as an important pathogen causing fruit rot in Korean apples (Lee et al., 2017). This study is the first study in the world to report that *Alternaria* and *Fusarium* species can co-infect apples and cause black spot symptoms. There are 18 species complexes and more than 300 *Fusarium* species. The morphological characteristics of *Fusarium* species are very similar, though with a large amount of minor morphological variability. It has also been difficult to identify the sexual stages of most *Fusarium* species, which causes many challenges for taxonomic identification (Thomas et al., 2019; Crous et al., 2021b). Accurate identification of *Fusarium*-induced plant diseases is a critical factor for effective scientific disease control (Jayawardena et al., 2021; Manawasinghe et al., 2021). By far the most informative and frequently used genes include *EF1-α*, *RPB1*, and *RPB2*, and they often accurately distinguish between *Fusarium* species (Crous et al., 2021b; Wang et al., 2022; O’Donnell et al., 2022). Over the past 25 years, genealogical concordance phylogenetic species recognition (GCPSR)-driven delimitation and identification of *Fusarium* species has largely been dependent on the nucleotide sequences of parts of phylogenetically informative protein-coding genes. By far the most informative and frequently used genes are *EF1-α*, *RPB1*, and *RPB2*, and they often accurately distinguish between *Fusarium* species (O’Donnell et al., 2022). In this study, a phylogenetic analysis (combining ITS, *EF1-α*, and *RPB2* gene sequences) showed that our seven representative *Fusarium* isolates from apples with bagged apple black spot disease clustered in the same branch as representative *F. acuminatum* isolates from GenBank (with 99% branch support rate).

## 5 Conclusion

In summary, this study revealed that bagged apple black spot disease during apple harvesting from September to October 2023 in northern Shaanxi is caused by *A. alternata* and *F. acuminatum*, with *A. alternata* being the dominant species. The pathogenicity of these two species was confirmed on detached apple fruits as single inoculations and co-inoculations. *F. acuminatum* is more pathogenic than *A. alternate*, while these two species infect together the disease severity is higher than a single infection. The results of this study provide an important foundation for understanding the epidemics of bagged apple black spot disease and formulating chemical control measures in northern Shaanxi Province. However, additional research is needed to clarify the roles of these fungi and their relationship with apple black spot disease during the whole apple-growing season.

## Data Availability

The datasets presented in this study can be found in online repositories. The names of the repository/repositories and accession number(s) can be found in the article/Supplementary material.
