# Supplementary material for: Identification and pathogenicity of Alternaria and Fusarium species associated with bagged apple black spot disease in Shaanxi, China
Source: Front Microbiol. 2024 Sep 12;15:1457315. doi: 10.3389/fmicb.2024.1457315 (PMC11424465; doi:10.3389/fmicb.2024.1457315)
Supplement: Supplementary file 1 [file Data_Sheet_1.ZIP › Tables.docx]

**Table1.** Summary of published universal primers used for *Fusarium* and *Alternaria* species determination associated with bagged apple black spot in Shaanxi, China.

| Gene/Locus | Primer name | Primer sequences (5’-3’)* | PCR amplication on procedures | Reference |
| --- | --- | --- | --- | --- |
| ITS | ITS1 | TCCGTAGGTGAACCTGCGG | 94°C 5 min; 30 cycles of 94°C 1 min, 55°C 30 s, 72°C 1 min; 72°C 10 min | (Woudenberg et al., 2014) |
|  | ITS4 | TCCTCCGCTTATTGATATGC |  |  |
| TEF1-α | EF1-728F | CATCGAGAAGTTCGAGAAGG | 95°C 3 min; 35 cycles of 94°C 30 s, 55°C 30 s, 72°C 1 min; 72°C 10 min | (Carbone and Kohn, 1999) |
|  | EF1-986R | TACTTGAAGGAACCCTTACC |  |  |
| endoPG | PG3 | TACCATGGTTCTTTCCGA | 94°C 5 min; 35 cycles of 94°C 45 s, 56°C 45 s, 72°C 45 s; 72°C 7 min | (Andrew et al., 2009a) |
|  | PG2b | GAGAATTCRCARTCRTCYTGRTT |  |  |
| OPA1-3 | OPA1-3L | CAGGCCCTTCCAATCCAT | 94°C 5 min; 35 cycles of 94°C 45 s, 58°C 45 s, 72°C 45 s; 72°C 7 min | (Peever et al., 2004) |
|  | OPA1-3R | AGGCCCTTCAAGCTCTCTTC |  |  |
| RPB2 | RPB2-5F2 | GAYGAYMGWGATCAYTTYGG | 94°C 5 min; 5 cycles of 94°C 45 s, 60°C 45 s, 72°C 2 min; 5 cycles of 94°C 45 s, 58°C 45 s, 72°C 2 min; 30 cycles of 94°C 45 s, 54°C 45 s, 72°C 2 min; 72°C 7 min | (Woudenberg et al., 2014) |
|  | fRPB2-7cR | CCCATRGCTTGYTTRCCCAT |  |  |

*Y = T or C; M = A or C; W = A or T; R = A or G.

**Table 2** *Alternaria* and *Fusarium* isolates obtained in this study and downloaded from GenBank with accession numbers used for phylogenetic analyses^y^

|  |  |  | GenBank accession No. | | | | |
| --- | --- | --- | --- | --- | --- | --- | --- |
| Isolate | Species | origin | ITS | EF1-α | RPB2 | endoPG | OPA1-3 |
| **WHWNSHJ4** | ***F. acuminatum*** | **Baota** | **PP336551** | **PP351904** | **－** | **－** | **－** |
| **WHWNSHJ5** | ***F. acuminatum*** | **Baota** | **PP336552** | **PP351905** | **PP351911** | **－** | **－** |
| **WHWNSHJ1** | ***F. acuminatum*** | **Baota** | **PP336553** | **PP351906** | **PP351912** | **－** | **－** |
| **YICASTK7** | ***F. acuminatum*** | **Yichuan** | **PP336554** | **PP351907** | **PP351913** | **－** | **－** |
| **YICASTK4** | ***F. acuminatum*** | **Yichuan** | **PP336555** | **PP351908** | **－** | **－** | **－** |
| **YICASTK3** | ***F. acuminatum*** | **Yichuan** | **PP336556** | **PP351909** | **－** | **－** | **－** |
| **YICASTK12** | ***F. acuminatum*** | **Yichuan** | **PP336557** | **PP351910** | **PP351914** | **－** | **－** |
| NL19-077002^*^ | *F. acuminatum* | Netherlands | MZ890557 | MZ921910 | MZ921779 | － | － |
| F201136 | *F. acuminatum* | China | KM527098 | KM527106 | KM520372 | － | － |
| CYF017 | *F. proliferatum* | China | MG384385 | MG674276 | MK027419 | － | － |
| CYF035 | *F. proliferatum* | China | MG384386 | MG674279 | MK027420 | － | － |
| CBS 101427^*^ | *F. solani* | USA | EU329691 | DQ246834 | － | － | － |
| NRRL 29132^*^ | *F. solani* | Germany | DQ094388 | DQ246915 | － | － | － |
| P55HS | *F. tricinctum* | Poland | － | MZ078998 | MZ078959 | － | － |
| CBS 119173^*^ | *F. graminearum* | Poland | － | KT855178 | KT855204 | － | － |
| NRRL 20697^*^ | *F. equiseti* | USA | GQ505683 | GQ505594 | JX171595 | － | － |
| CBS 632.76^*^ | *F. lunatum* | Germany | EU926224 | EU926291 | － | － | － |
| **SWHBS4-2** | ***A. alternata*** | **Baota** | **PP346356** | **PP351930** | **PP351921** | **PP351915** | **PP376078** |
| **YICWNSHJ9** | ***A. alternata*** | **Yichuan** | **PP346360** | **PP351933** | **PP351925** | **PP351917** | **PP376082** |
| **YICWNSHJ7** | ***A. alternata*** | **Yichuan** | **PP346361** | **PP351934** | **PP351926** | **PP351918** | **PP376083** |
| **YICASTK5** | ***A. alternata*** | **Yichuan** | **PP346363** | **PP351936** | **PP351922** | **PP351920** | **PP376085** |
| CBS 106.24^*^ | *A. alternata* | USA | － | KP125073 | KP124766 | AY295020 | － |
| CBS 104.26 | *A. alternata* | Unknown | － | KP125074 | KP124767 | KP123995 | － |
| CH-37 | *A. alternata* | Italy | － | － | OP899773 | OP899733 | － |
| 9-1 | *A. alternata* | China | － | － | － | OK428544 | － |
| A10 | *A. alternata* | China | － | － | － | MN894680 |  |
| CBS 118486^*^ | *A. iridiaustralis* | Australia | － | KP125214 | KP124905 | KP124140 | － |
| CBS 878.95 | *A. jacinthicola* | Mauritius | － | KP125216 | KP124907 | KP124142 | － |
| CBS 133751^*^ | *A. jacinthicola* | Mali | － | KP125217 | KP124908 | KP124143 | － |
| CBS 113.35 | *A. longipes* | Unknown | － | KP125219 | KP124910 | KP124145 | － |
| CBS 539.94 | *A. longipes* | USA | － | KP125220 | KP124911 | KP124146 | － |
| CBS 118809^*^ | *A. alstroemeriae* | Australia | － | KP125072 | KP124765 | KP123994 | － |
| CBS 107.38^*^ | *A. burnsii* | India | － | KP125198 | KP124889 | KP124124 | － |
| CBS 489.92^*^ | *A. eichhorniae* | India | － | KP125204 | KP124895 | KP124130 | － |
| EGS 90-0512 | *A. gaisen* | Japan | － | KC584658 | KC584399 | AY295033 | － |
| CBS 102605^*^  CBS 109730 | *A.arborescens*  *A.arborescens* | USA  USA | －  － | KC584636  KP125177 | KC584377  KP124869 | AY295028  KP124103 | －  － |

^y^ All the ex-type isolates used in this study are marked by an asterisk(*). The isolates in this study are indicated in bold font.
