# Supplementary figures and images for: Identification and pathogenicity of Alternaria and Fusarium species associated with bagged apple black spot disease in Shaanxi, China
Source: Front Microbiol. 2024 Sep 12;15:1457315. doi: 10.3389/fmicb.2024.1457315 (PMC11424465; doi:10.3389/fmicb.2024.1457315)

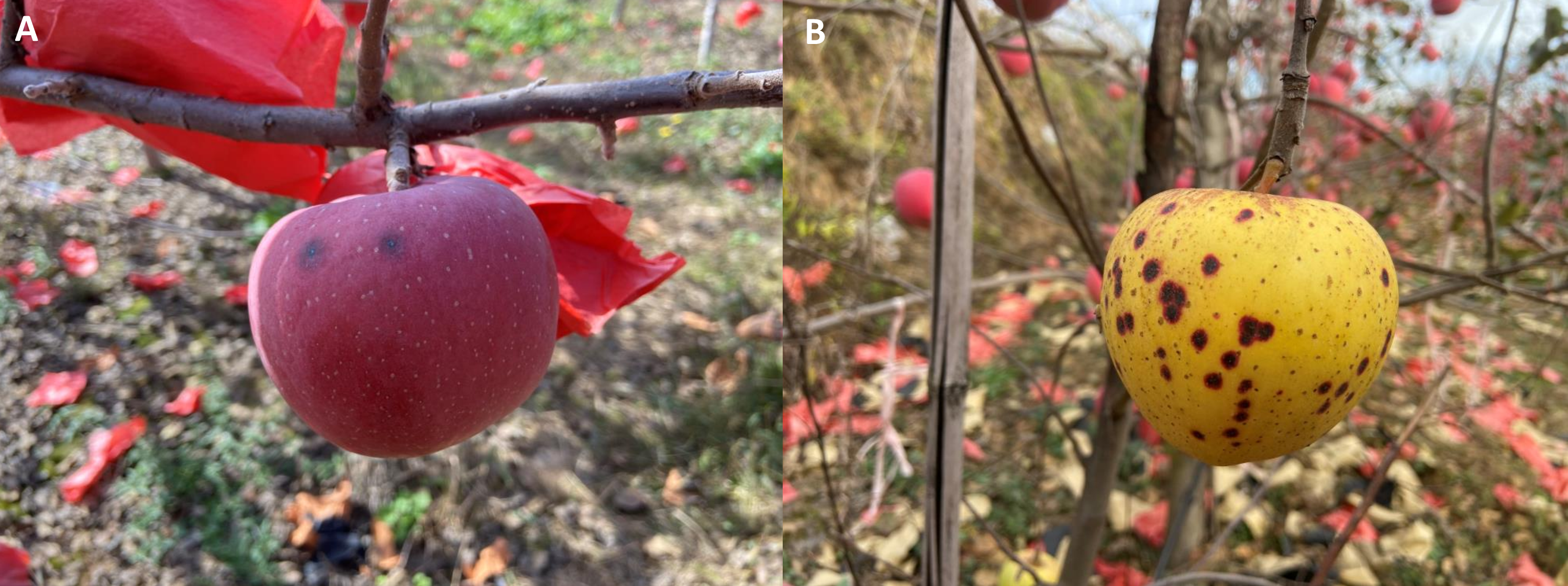

Supplement: Supplementary file 1 [file Data_Sheet_1.ZIP › FIGURE 1.tiff]

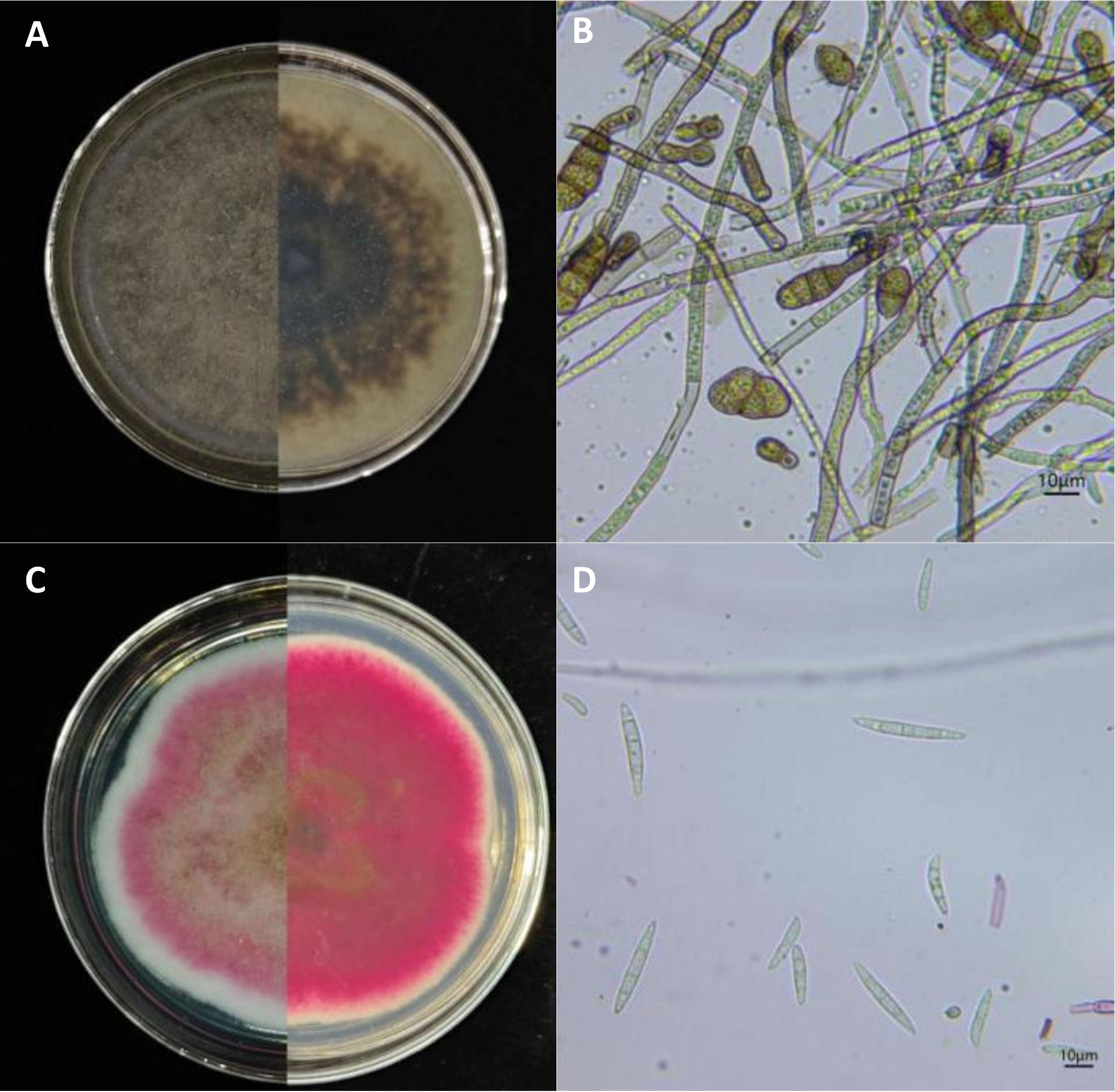

Supplement: Supplementary file 1 [file Data_Sheet_1.ZIP › FIGURE 2.tiff]

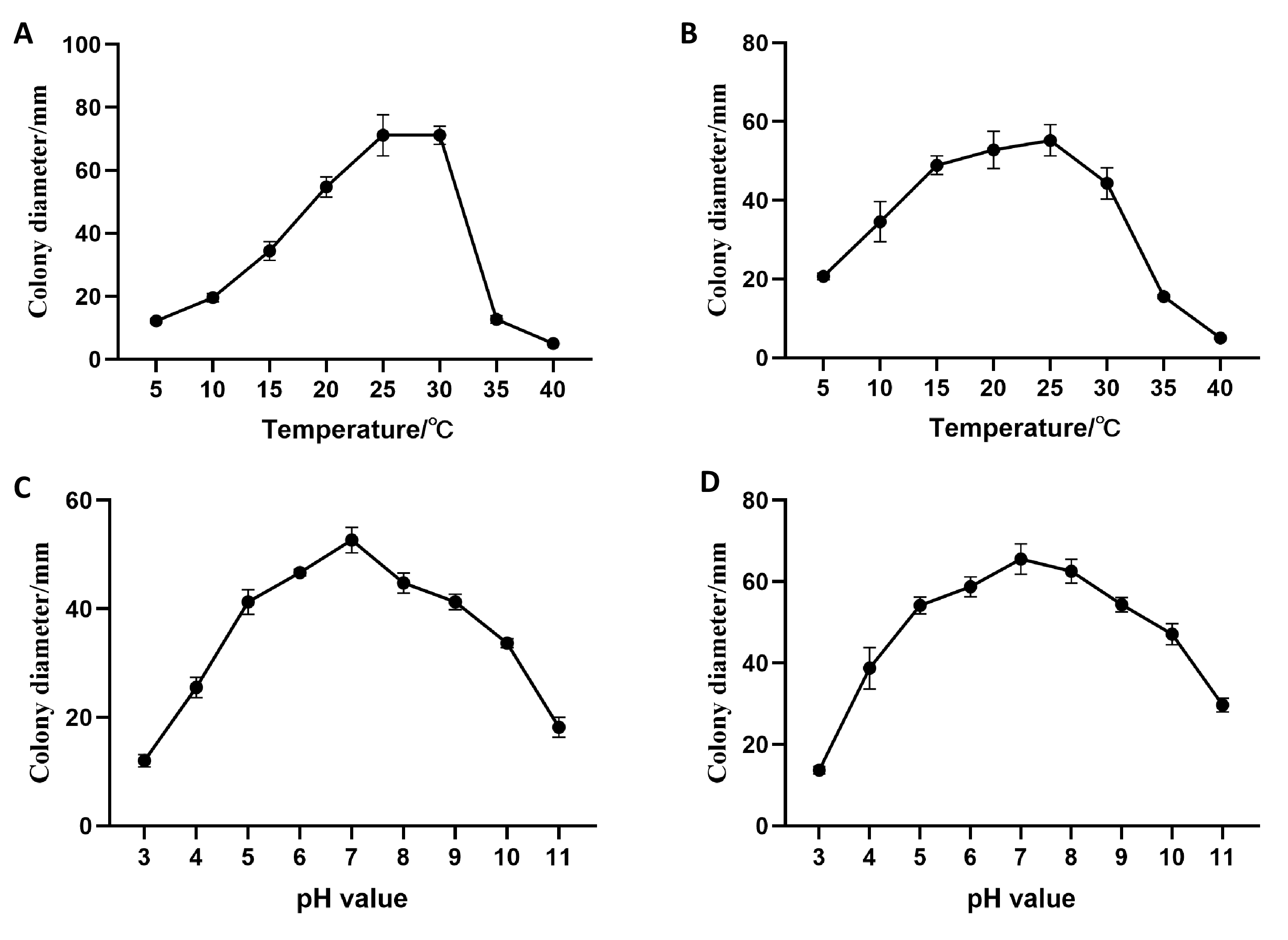

Supplement: Supplementary file 1 [file Data_Sheet_1.ZIP › FIGURE 5.tiff]

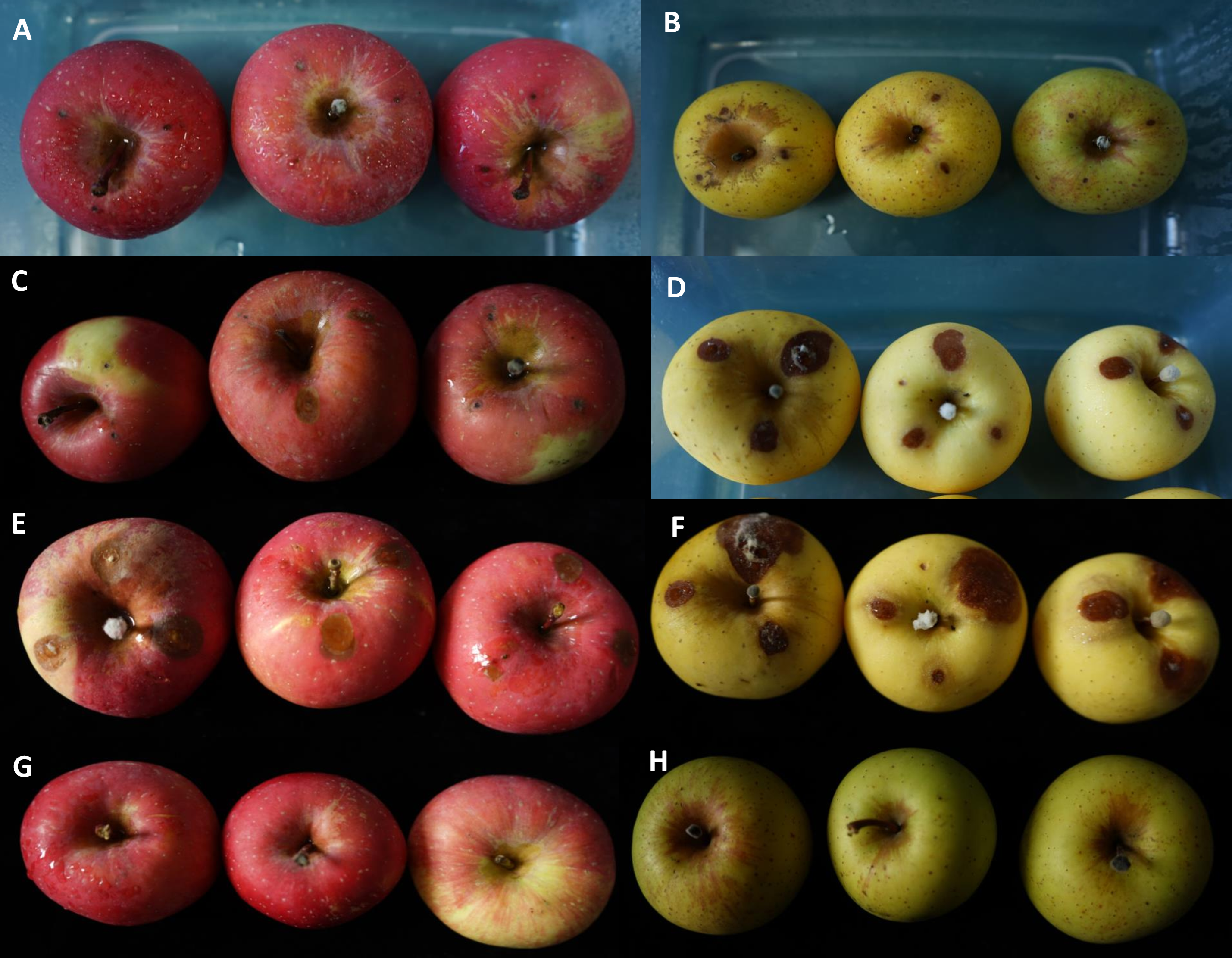

Supplement: Supplementary file 1 [file Data_Sheet_1.ZIP › FIGURE 6.tiff]

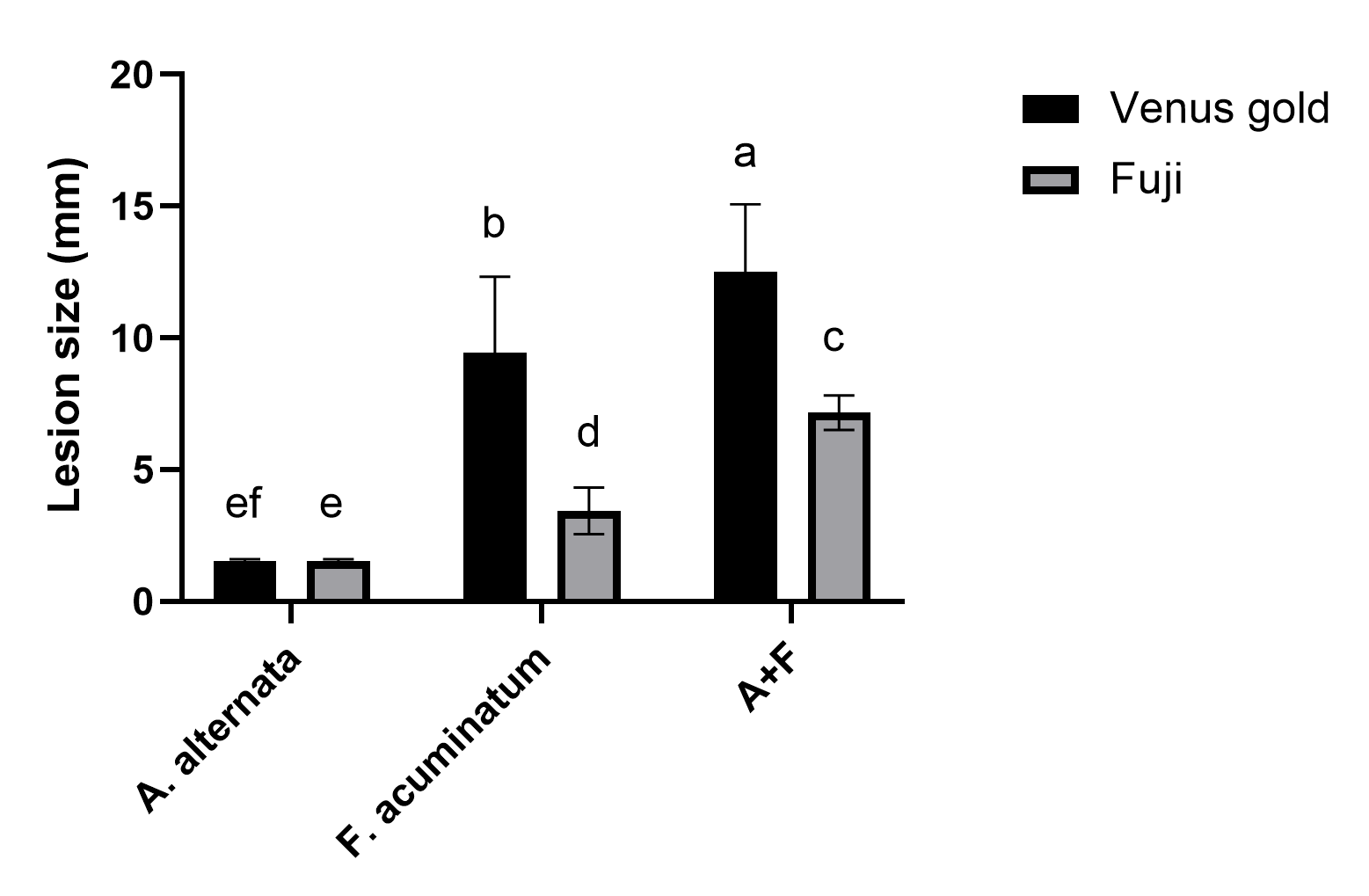

Supplement: Supplementary file 1 [file Data_Sheet_1.ZIP › FIGURE 7.tif]
